# The Effects of Topical Application of Polycal (a 2:98 (g/g) Mixture of Polycan and Calcium Gluconate) on Experimental Periodontitis and Alveolar Bone Loss in Rats

**DOI:** 10.3390/molecules21040527

**Published:** 2016-04-22

**Authors:** Sang-In Park, Su-Jin Kang, Chang-Hyun Han, Joo-Wan Kim, Chang-Hyun Song, Sang-Nam Lee, Sae-Kwang Ku, Young-Joon Lee

**Affiliations:** 1 The Medical Research Center for Globalization of Herbal Medicine, Daegu Haany University, Gyeongsan 38610, Korea; sangin0118@naver.com (S.-I.P.); vegonia1@hanmail.net (S.-J.K.); dvmsong@hotmail.com (C.-H.S.); 2 Department of Histology and Anatomy, College of Korean Medicine, Daegu Haany University, Gyeongsan 38610, Korea; 3 Department of Preventive Medicine, College of Korean Medicine, Deagu Haany University, Gyeongsan 38610, Korea; 4 Clinical Research Division, Korean Institute of Oriental Medicine, Daejeon 34054, Korea; chhan@kiom.re.kr; 5 ARIBIO, Jecheon 27159, Korea; kimjw@aribio.com; 6 Department of Qigong, College of Korean Medicine, Deagu Haany University, Gyeongsan 38610, Korea; lsn1974@dhu.ac.kr

**Keywords:** alveolar bone loss, anti-bacterial effects, anti-inflammatory, antioxidant, β-glucan, calcium gluconate

## Abstract

The aim of this study was to observe whether Polycal has inhibitory activity on ligation-induced experimental periodontitis and related alveolar bone loss in rats following topical application to the gingival regions. One day after the ligation placements, Polycal (50, 25, and 12.5 mg/mL solutions at 200 μL/rat) was topically applied to the ligated gingival regions daily for 10 days. Changes in bodyweight, alveolar bone loss index, and total number of buccal gingival aerobic bacterial cells were monitored, and the anti-inflammatory effects were investigated via myeloperoxidase activity and levels of the pro-inflammatory cytokines IL-1β and TNF-α. The activities of inducible nitric oxide synthase (iNOS) and lipid peroxidation (MDA) were also evaluated. Bacterial proliferation, periodontitis, and alveolar bone loss induced by ligature placements were significantly inhibited after 10 days of continuous topical application of Polycal. These results indicate that topical application of Polycal has a significant inhibitory effect on periodontitis and related alveolar bone loss in rats mediated by antibacterial, anti-inflammatory, and anti-oxidative activities.

## 1. Introduction

Periodontitis is an important cause of tooth loss in adults [1]. It is a chronic inflammatory disease that is characterized by localized bone resorption [2,3]. The presence of bacterial plaques is considered as the main pathogenesis involved in periodontitis, which may initiate a localized inflammatory reaction [4]. These inflammatory responses lead to edema, leukocyte infiltration, and the release of inflammatory mediators, causing periodontal pocket formation, connective tissue detachment, and alveolar bone resorption, and ultimately leading to tooth loss [3,5]. Recently, nitric oxide activity and oxidative stress have been shown to be important in the pathogenesis of periodontitis [6], and many antioxidants have been shown to exhibit favorable effects on periodontitis and related alveolar bone loss [7,8].

Polycan is a purified β-glucan from *Aureobasidium pullulans*, which is mainly comprised of β-1,3/1,6-glucan, together with other organic materials, including amino acids, mono- or di-unsaturated fatty acids, and fibrous polysaccharides [9]. Recently, we showed that Polycan has anti-osteoporotic effects [10,11], inhibiting bone loss and accelerating bone formation. Polycan also has anti-inflammatory effects on xylene-induced acute inflammation [12], as well as formalin-induced chronic inflammation [13]. It has also been shown to have favorable effects on the prevention and treatment of cisplatin-induced kidney damage [14], as well as ligation-induced periodontitis and related alveolar bone loss [8], mediated via anti-oxidant and anti-inflammatory mechanisms. Calcium salts have been shown to exhibit anti-inflammatory activity [15,16,17]. The anti-inflammatory activity of calcium gluconate has been shown to enhance that of non-steroidal anti-inflammatory drugs [18], as well as to mitigate ligation-induced periodontitis and related alveolar bone loss [7] and collagen-induced rheumatoid arthritis [19] via anti-oxidant and anti-inflammatory mechanisms.

Mixtures of Polycan and calcium gluconate have been shown to have synergic anti-osteoporotic effects in ovariectomized rats [20] with low toxicity [21]. Oral administration of Polycal (a 2:98 (g/g) mixture of Polycan and calcium gluconate) has favorable synergic effects on ligation-induced periodontitis, demonstrating potential as a candidate component in the prevention of periodontal disease [22]; however, it remains unclear whether this mixture also has favorable effects following topical application to the gingival regions.

## 2. Results

### 2.1. Changes in Bodyweight

**Figure 1:**
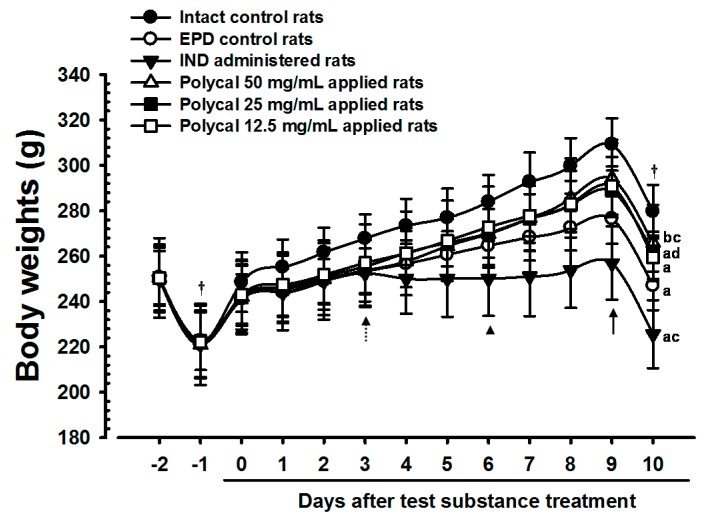
Body weights changes in Intact or EPD Rats. Note that significant (*p* < 0.01 or *p* < 0.05) decreases of body weights were detected in EPD control from four days after ligature placement (from Day 3 after initiation of administration) as compared with Intact control (dot arrow). However, the body weights of all three concentrations of Polycal topical applied rats were markedly increased as compared with EPD control from nine days after start of administration (arrow). In contrary, significant (*p* < 0.01 or *p* < 0.05) decreases of body weights were detected in IND 5 mg/kg orally administered rats from six days after initiation of administration as compared with EPD control (arrowhead), in this experiment. Values are expressed mean ± S.D. of eight rats. EPD = Experimental periodontal diseases induced by ligature placement around the cervix of upper left incisor teeth; Polycal = Polycan and calcium gluconate 2:98 (g/g) mixture, which was topically applied to ligated gingival regions, once a day for 10 days (200 μL/rats); IND = Indomethacin, which was orally administered at 5 mg/kg dose level, once a day for 10 days; The day −1 means the day of ligature placement. All rats were overnight fasted before ligature placement and sacrifice, respectively (†). ^a^ *p <* 0.01 and ^b^ *p <* 0.05 as compared with Intact control; ^c^ *p <* 0.01 and ^d^ *p <* 0.05 as compared with EPD control.

The bodyweight of rats treated with all three concentrations of Polycal exhibited marked increases compared with the EPD control from nine days after start of administration. Furthermore, the gain in bodyweight during the 10-day administration period was concentration response trend, and increased significantly in rats treated with higher concentrations of Polycal. By contrast, a significant decrease in bodyweight was found in the IND group from six days after the start of administration compared with the EPD control (Table 1, Figure 1).

### 2.2. Alveolar Bone Loss Scores

**Figure 2:**
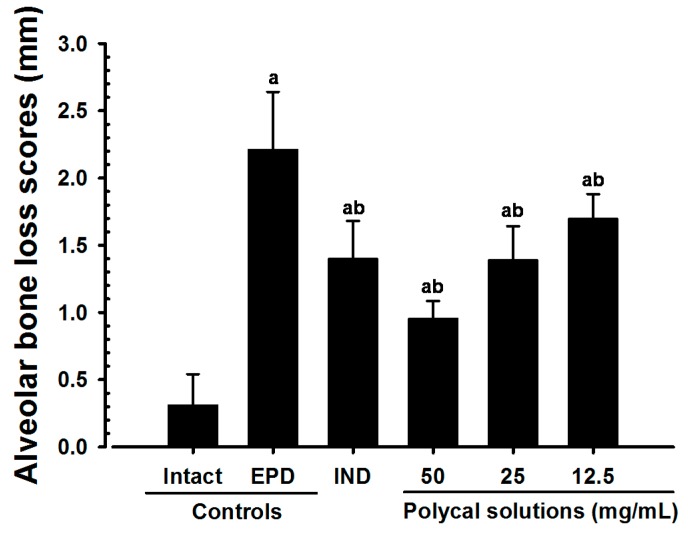
Alveolar Bone Loss Scores in Intact or EPD Rats. Note that significant increases of exposed teeth roots areas and the alveolar bone loss scores were detected in EPD control as compared with Intact control. However, significant decreases of alveolar bone loss scores were detected in IND 5 mg/kg orally administrated rats and also in all three different concentrations of Polycal topically applied rats as compared with EPD control, respectively. Especially, all three different concentrations of Polycal topical applied rats showed decreases of bone loss scores as compared with EPD control rats. Values are expressed mean ± S.D. of eight rats, mm. EPD = experimental periodontal diseases induced by ligature placement around the cervix of upper left incisor teeth; Polycal = Polycan and calcium gluconate 2:98 (g/g) mixture, which was topically applied to ligated gingival regions, once a day for 10 days (200 μL/rats); IND = Indomethacin, which was orally administered at 5 mg/kg dose level, once a day for 10 days. ^a^ *p <* 0.01 as compared with Intact control; ^b^ *p <* 0.01 as compared with EPD control.

Significant increases in exposure of the tooth root were found in the EPD control compared with the Intact control. However, significantly lower alveolar bone loss scores were found in the IND group than in the EPD control, as well as in rats administered with Polycal. In particular, rats treated with Polycal showed a reduction in bone loss scores compared with the EPD control (Figure 2).

### 2.3. Gingival Viable Bacteria Counts

**Figure 3:**
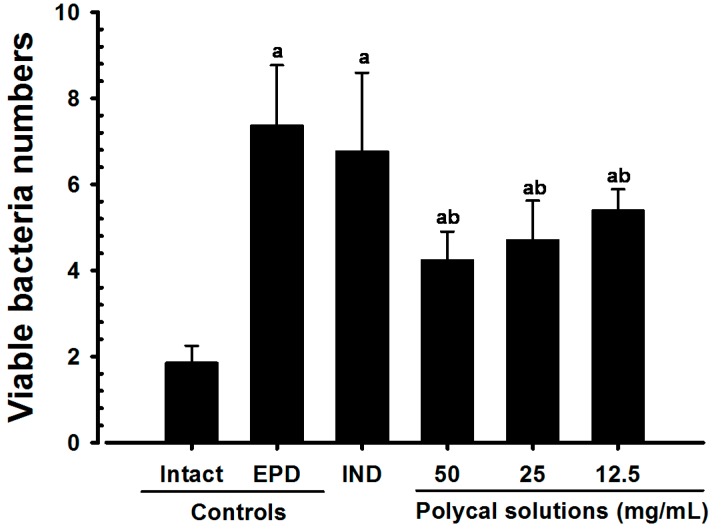
Gingival viable bacteria counts in Intact or EPD Rats. Note that significant increases of gingival viable bacteria numbers were detected in EPD control as compared with Intact control. However, significant decreases of viable bacteria numbers were detected in all three different concentrations of Polycal topical applied rats as compared with EPD control, respectively. Anyway, no meaningful changes on the viable bacteria numbers were demonstrated in IND 5 mg/kg oral administered rats as compared with EPD control, in this result. Values are expressed mean ± S.D. of eight rats, Log CFU × 10^5^/g tissue. EPD = experimental periodontal diseases induced by ligature placement around the cervix of upper left incisor teeth; Polycal = Polycan and calcium gluconate 2:98 (g/g) mixture, which was topically applied to ligated gingival regions, once a day for 10 days (200 μL/rats); IND = Indomethacin, which was orally administered at 5 mg/kg dose level, once a day for 10 days. ^a^ *p <* 0.01 as compared with Intact control; ^b^ *p <* 0.01 as compared with EPD control.

Significant increases in the number of viable gingival bacterial cells were detected in the EPD control group compared with the Intact control. However, significant decreases in the number of viable bacterial cells were found in the Polycal groups compared with the EPD control. No significant changes in the number of viable bacterial cells were demonstrated in the IND group compared with the EPD control (Figure 3).

### 2.4. Anti-Inflammatory and Anti-Oxidant Effects

Significant increase in the gingival MPO activity, IL-1β, TNF-α and MDA levels and iNOS activity were detected in the EPD control compared with the Intact control. However, compared with the EPD control, significant decreases in these indices were detected in rats treated with all test substances. In particular, rats treated with Polycal showed decrease in these indices in the buccal gingival tissues around the ligation compared with the EPD control rats.

#### 2.4.1. Changes on the Gingival MPO Activities

**Figure 4:**
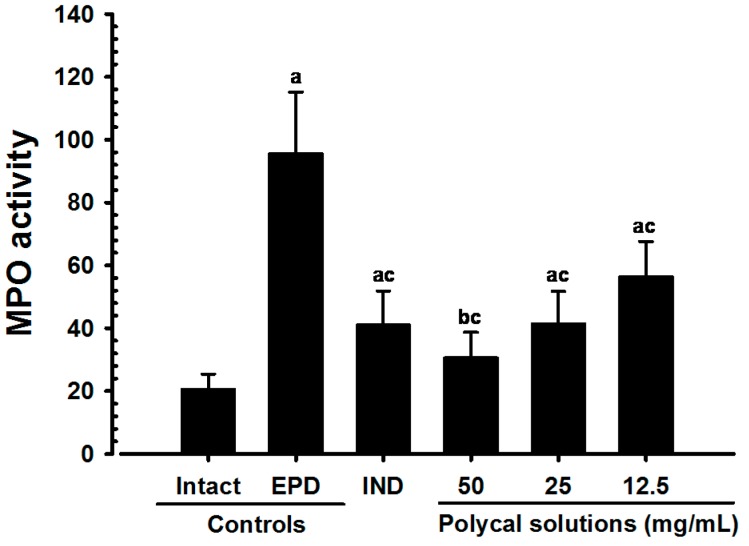
Gingival MPO activities in Intact or EPD rats. Values are expressed mean ± S.D. of eight rats, U/mg tissue. EPD = experimental periodontal diseases induced by ligature placement around the cervix of upper left incisor teeth; Polycal = Polycan and calcium gluconate 2:98 (g/g) mixture, which was topically applied to ligated gingival regions, once a day for 10 days (200 μL/rats); IND = Indomethacin, which was orally administered at 5 mg/kg dose level, once a day for 10 days. MPO = Myeloperoxidase. ^a^ *p <* 0.01 and ^b^ *p <* 0.05 as compared with Intact control; ^c^ *p <* 0.01 as compared with EPD control.

Significant (*p* < 0.01) decreases of gingival MPO activities were detected in IND 5 mg/kg orally administrated rats and also in all three different concentrations of Polycal topically applied rats, as compared with EPD control. Especially, all three different concentrations of Polycal topical applied rats showed decreases of gingival MPO activities in the buccal gingival tissues around ligation placed as compared with EPD control rats (Figure 4).

#### 2.4.2. Changes on the Gingival IL-1β Levels

**Figure 5:**
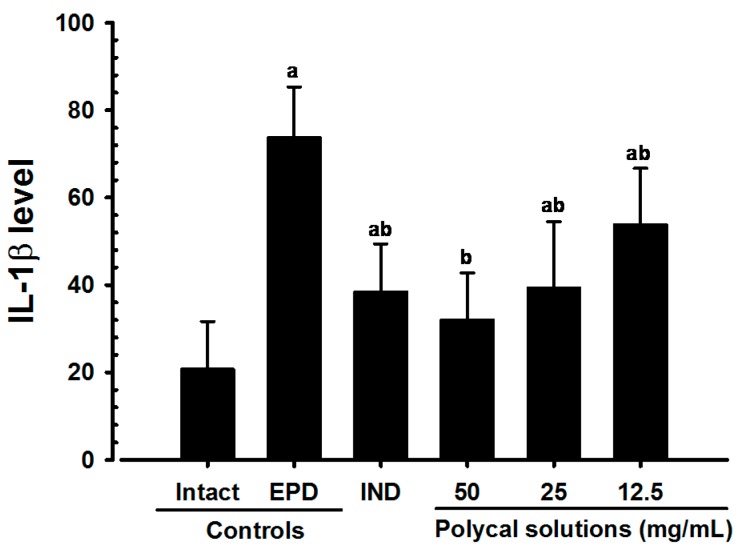
Gingival IL-1β levels in Intact or EPD rats. Values are expressed mean ± S.D. of eight rats, pg/mL. EPD = experimental periodontal diseases induced by ligature placement around the cervix of upper left incisor teeth; Polycal = Polycan and calcium gluconate 2:98 (g/g) mixture, which was topically applied to ligated gingival regions, once a day for 10 days (200 μL/rats); IND = Indomethacin, which was orally administered at 5 mg/kg dose level, once a day for 10 days. IL = Interleukin. ^a^ *p <* 0.01 as compared with Intact control; ^b^ *p <* 0.01 as compared with EPD control.

Significant (*p* < 0.01) decreases of gingival IL-1β levels were detected in all test substance treated rats as compared with EPD control. Especially, all three different concentrations of Polycal topical applied rats showed decreases of gingival IL-1β levels in the buccal gingival tissues around ligation placed as compared with EPD control rats (Figure 5).

#### 2.4.3. Changes on the Gingival TNF-α Levels

**Figure 6:**
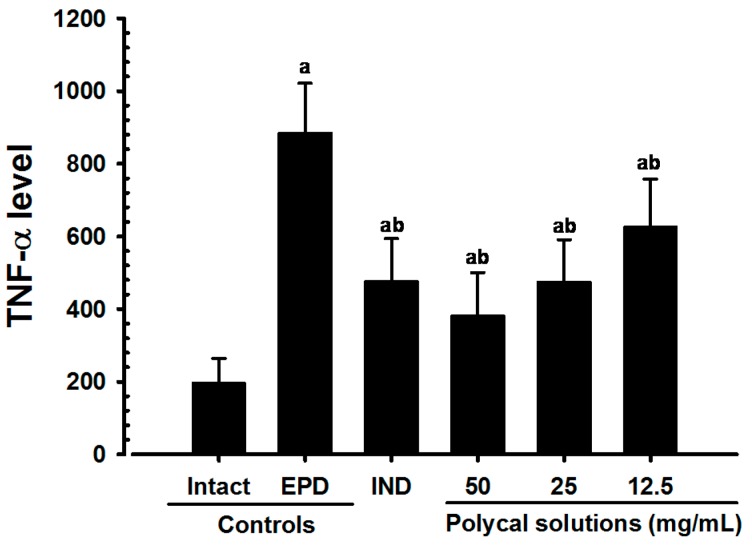
Gingival TNF-α Levels in Intact or EPD Rats. Values are expressed mean ± S.D. of eight rats, pg/mL. EPD = experimental periodontal diseases induced by ligature placement around the cervix of upper left incisor teeth; Polycal = Polycan and calcium gluconate 2:98 (g/g) mixture, which was topically applied to ligated gingival regions, once a day for 10 days (200 μL/rats). IND = Indomethacin, which was orally administered at 5 mg/kg dose level, once a day for 10 days; TNF = Tumor necrosis factor. ^a^ *p <* 0.01 as compared with Intact control; ^b^ *p <* 0.01 as compared with EPD control.

Significant (*p* < 0.01) decreases of gingival TNF-α levels were detected in all test substance treated rats including IND 5 mg/kg oral administered rats as compared with EPD control, respectively. Especially, all three different concentrations of Polycal topical applied rats showed decreases of gingival TNF-α levels in the buccal gingival tissues around ligation placed as compared with EPD control rats (Figure 6).

#### 2.4.4. Changes on the Gingival MDA Levels

**Figure 7:**
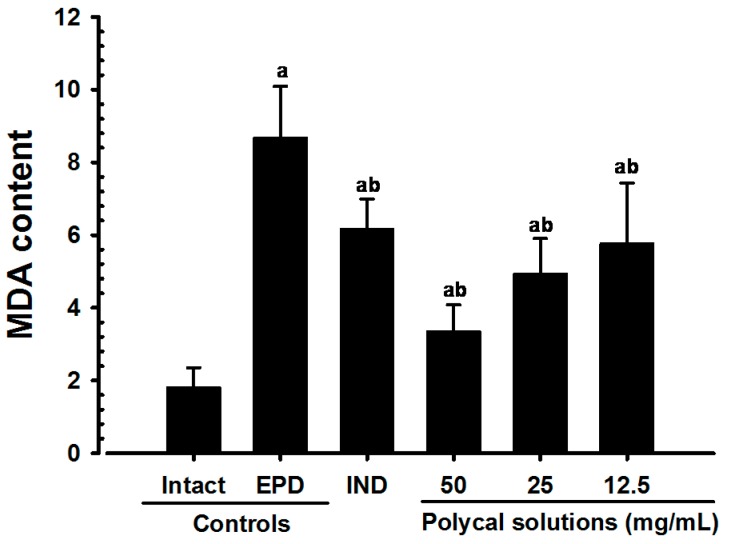
Gingival MDA levels in Intact or EPD Rats. Values are expressed mean ± S.D. of eight rats, μM/mg tissue. EPD = experimental periodontal diseases induced by ligature placement around the cervix of upper left incisor teeth; Polycal = Polycan and calcium gluconate 2:98 (g/g) mixture, which was topically applied to ligated gingival regions, once a day for 10 days (200 μL/rats). IND = Indomethacin, which was orally administered at 5 mg/kg dose level, once a day for 10 days; MDA = Malondialdehyde. ^a^ *p <* 0.01 as compared with Intact control; ^b^ *p <* 0.01 as compared with EPD control.

Significant (*p* < 0.01) decreases of gingival MDA levels were detected in all test substance treated rats including Polycal 50 mg/mL concentration solution topical applied rats as compared with EPD control. Especially, all three different concentrations of Polycal topical applied rats showed decreases of gingival MDA levels in the buccal gingival tissues around ligation placed as compared with EPD control rats (Figure 7).

#### 2.4.5. Changes on the Gingival iNOS Activities

**Figure 8:**
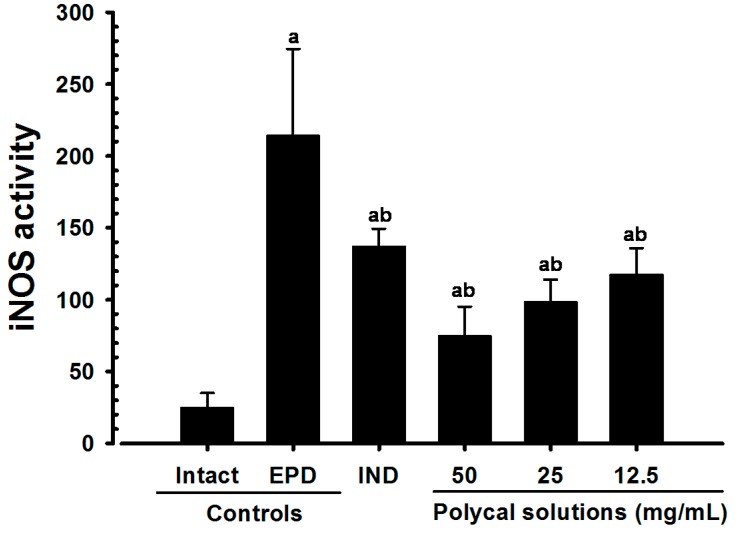
Gingival iNOS activities in Intact or EPD rats. Values are expressed mean ± S.D. of eight rats, fM/mg tissue/min. EPD = experimental periodontal diseases induced by ligature placement around the cervix of upper left incisor teeth; Polycal = Polycan and calcium gluconate 2:98 (g/g) mixture, which was topically applied to ligated gingival regions, once a day for 10 days (200 μL/rats); IND = Indomethacin, which was orally administered at 5 mg/kg dose level, once a day for 10 days; iNOS = Inducible nitric oxide synthase. ^a^ *p <* 0.01 as compared with Intact control; ^b^ *p <* 0.01 as compared with EPD control.

Significant (*p* < 0.01) decreases of gingival iNOS activities were detected in all test substance treated rats including IND 5 mg/kg oral administered rats as compared with EPD control, respectively. Especially, all three different concentrations of Polycal topical applied rats showed decreases of gingival iNOS activities in the buccal gingival tissues around ligation placed as compared with EPD control rats (Figure 8).

### 2.5. Histopathology of the Maxillary Regions

**Figure 9:**
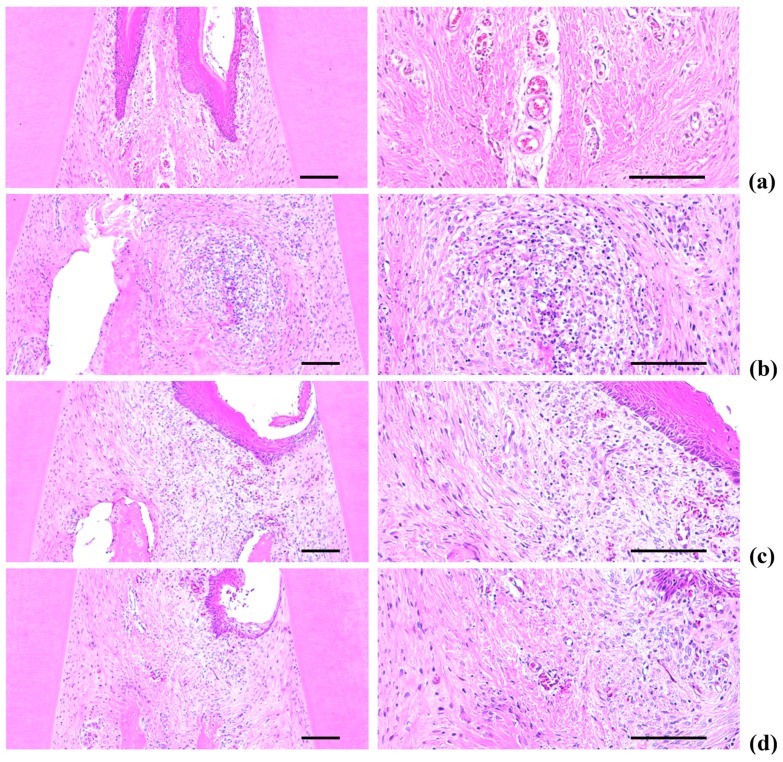

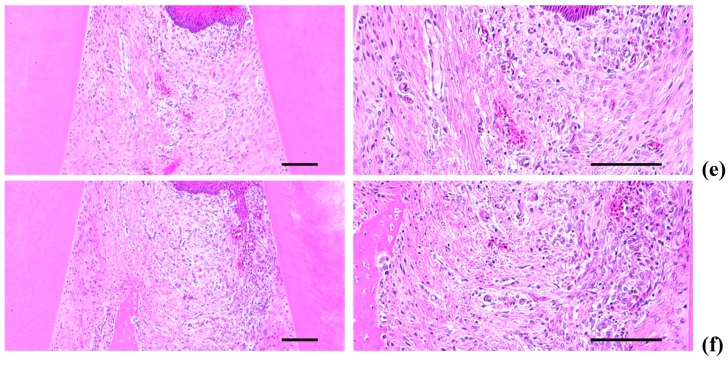
Representative histological images of gingival tissues between upper incisor teeth, taken from Intact or EPD rats around upper left incisor teeth: (**a**) Intact control (Normal control, non-ligated and distilled water topically applied rats); (**b**) EPD control (Ligated EPD induced and distilled water topically applied rats); (**c**) IND (Ligated EPD induced and indomethacin 5 mg/kg orally administered rats); (**d**) Polycal-H (Ligated EPD induced and Polycal 50 mg/mL solution topically applied rats); (**e**) Polycal-M (Ligated EPD induced and Polycal 25 mg/mL solution topically applied rats); and (**f**) Polycal-L (Ligated EPD induced and Polycal 12.5 mg/mL solution topically applied rats). All H & E stain. Scale bars = 120 µm.

**Figure 10:**
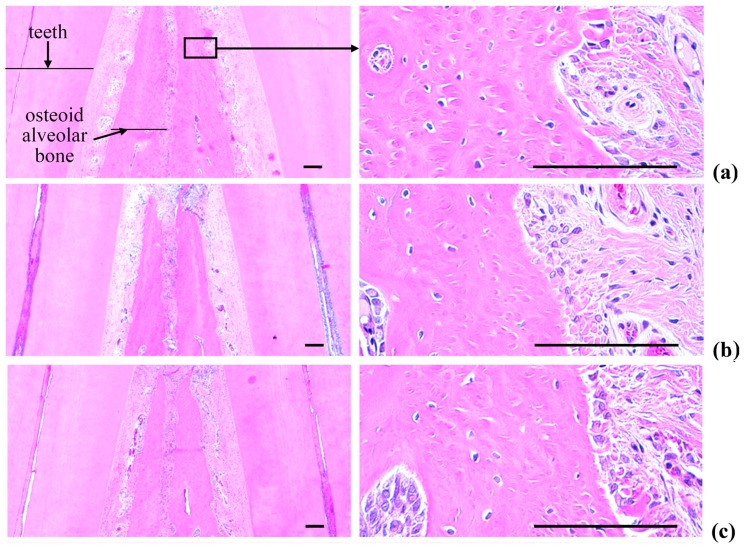

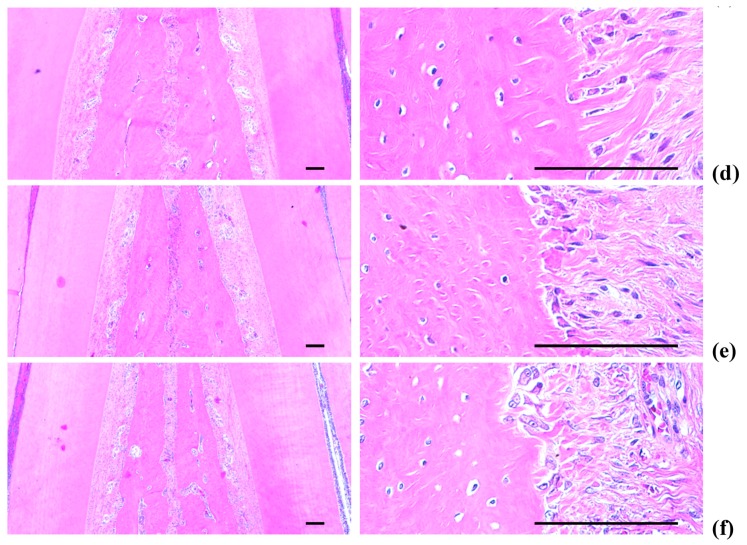
Representative histological images of alveolar bone areas between the upper incisor teeth, taken from Intact or EPD rats around upper left incisor teeth: (**a**) Intact control (Normal control, non-ligated and distilled water topically applied rats); (**b**) EPD control (Ligated EPD induced and distilled water topically applied rats); (**c**) IND (Ligated EPD induced and indomethacin 5 mg/kg orally administered rats); (**d**) Polycal-H (Ligated EPD induced and Polycal 50 mg/mL solution topically applied rats); (**e**) Polycal-M (Ligated EPD induced and Polycal 25 mg/mL solution topically applied rats); and (**f**) Polycal-L (Ligated EPD induced and Polycal 12.5 mg/mL solution topically applied rats). All H & E stain. Scale bars = 120 µm.

Marked increases in inflammatory cell infiltration (mainly polymorphneutrophils (PMNs)) were found in the gingival tissues between the upper left and right incisors in the EPD control, including severe edematous changes; *i.e.*, loosening of the collagen fibers and a loss of compactness. We also observed increased activation of osteoclast cells, as well as increases in the number and percentage of regions occupied by osteoclasts on the alveolar bone surface (OS/BS) in the alveolar bone areas of the EPD control, accompanied by a marked decrease in the volume of osteoid alveolar bone. It follows that periodontitis and related alveolar bone loss was induced by the ligature placements. However, the histopathological evidence for periodontitis, as well as the infiltration of inflammatory cell, the loosening of collagen fiber, the activation of osteoclast cells, and the decrement of the volume of osteoid alveolar bone, was markedly reduced by oral treatment with IND, as well as by topical application of Polycal at all three concentrations compared with the EPD control rats (Table 2, Figure 9 and Figure 10).

#### 2.5.1. Changes on the Numbers of Inflammatory Cells Infiltrated in Gingival Tissues

Significant (*p* < 0.01) decreases of the numbers of inflammatory cells infiltrated in gingival tissues were detected in all test substance treated rats including IND 5 mg/kg oral administered rats as compared with EPD control, respectively. Especially, all three different concentrations of Polycal topical applied rats showed decreases of gingival inflammatory cells around ligation placed as compared with EPD control rats (Table 2, Figure 9).

#### 2.5.2. Changes on the Collagen Fiber Occupied Regions in Gingival Tissues

Significant (*p* < 0.01) increases of collagen fiber occupied regions in gingival tissues were detected in all test substance administered rats including Polycal 50 mg/mL concentration topically applied rats as compared with EPD control, respectively. Especially, all three different concentrations of Polycal topical applied rats showed increases of collagen fiber occupied regions in gingival tissues around ligation placed as compared with EPD control rats (Table 2, Figure 9).

#### 2.5.3. Changes on the Alveolar Bone Volumes

Significant (*p* < 0.01) increases of the alveolar bone volumes were detected in all test substance treated rats as compared with EPD control, respectively. Especially, all three different concentrations of Polycal topical applied rats showed increases of alveolar bone volumes between upper left and right incisor teeth where ligation was placed as compared with EPD control rats (Table 2, Figure 10).

#### 2.5.4. Changes on the Osteoclast Cells

Significant (*p* < 0.01) decreases of osteoclast cell numbers and OS/BS were detected in IND 5 mg/kg oral administrated rats and also in all three different concentrations of Polycal topical applied rats as compared with EPD control, respectively (Table 2, Figure 10).

### 2.6. Histological Scores

**Figure 11:**
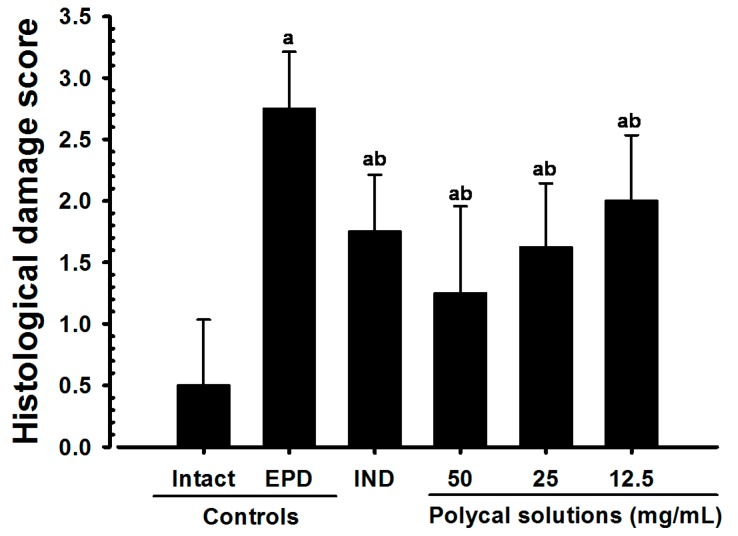
Histological scores in Intact or EPD rats. Values are expressed mean ± S.D. of eight rats, score (Max = 3). EPD = experimental periodontal diseases induced by ligature placement around the cervix of upper left incisor teeth; Polycal = Polycan and calcium gluconate 2:98 (g/g) mixture, which was topically applied to ligated gingival regions, once a day for 10 days (200 μL/rats); IND = Indomethacin, which was orally administered at 5 mg/kg dose level, once a day for 10 days. ^a^ *p* < 0.01 as compared with Intact control; ^b^ *p* < 0.01 as compared with EPD control.

Significant increases in histological scores were detected in the EPD control compared with the Intact control. However, marked decreases in histological scores were found in all the rats treated with all test substances, including Polycal, compared with the EPD control. In particular, the rats treated with all three concentrations of Polycal exhibited decreases in histological scores around the gingival and alveolar bone tissues close to the ligation placement compared with the EPD control (Figure 11).

## 3. Discussion

Periodontitis and the related alveolar bone loss due to EPD leads to malmastication, which results in a marked drop in bodyweight [4,23]. Therefore, lack of EPD-related loss in bodyweight can be considered indirect evidence of amelioration of periodontitis and related alveolar bone loss [8,22]. We found a significant decrease in the alveolar bone loss scores with oral administration of IND, as well as with topical application of all three concentrations of Polycal, where the response was concentration response trend. Additionally, notable increases in bodyweight with topically applied of Polycal were observed compared with the EPD control.

Bacteria are considered the primary etiologic agents of periodontal disease [24]. It has been reported that the periodontal microbial flora changes following the onset of periodontal disease in rats, whereby anaerobic Gram-negative bacilli predominate following ligation of the tooth cervix [4,5]. This is similar with the results of our work. However, significant decreases in the number of viable bacterial cells were found in rats treated with all three concentrations of Polycal. Both direct and indirect evidence of the antibacterial effects of β-glucan is well-documented [25,26,27]. In current study, the ligature model was selected to induce experimental periodontitis in rats. Dietary manipulation, introduction of pathogenic microorganism and placement of a ligature were used to induce experimental periodontitis. Placement of a ligature needs to special skills and is also highly predictable method for measuring the effects of drugs against periodontal diseases. However, because ligature-induced peritonitis is the mechanical injury caused during the placement of a ligature, the process of natural disease was different. Thus, profound studies were required to clarify the effect of Polycal against peritonitis disease induced by microorganism.

The importance of acute inflammatory cells on gingival tissue in the evolution of periodontal disease has been established [5,28]. It is well-known that oxygen metabolites are important in the recruitment of neutrophils (preferentially PMNs) into injured tissues [29]. MPO is an activating cytotoxic enzyme that is released from PMNs [30], levels of which increase in periodontal diseases [7,22]. A reduction of neutrophil influx into gingival tissues can be assessed by characterizing MPO activity [4]. We found that the increases of gingival MPO levels were significantly inhibited by treatment with IND and by topical application of all three concentrations of Polycal. In particular, the three concentrations of Polycal exhibited decreases in gingival MPO activity compared with the EPD control.

The cytokine TNF-α is associated with critical events leading to T-lineage commitment and differentiation [31]. TNF-α enhances proliferation of B and T cells, and promotes the generation of cytotoxic T cells. In addition, it enhances IL-2-induced immunoglobulin production, and augments IL-2-stimulated activity of natural killer cells, as well as proliferation of monocytes [32]. TNF-α may potentiate periodontitis by stimulating the release of eicosanoids, as well as other cytokines such as IL-1. There are two forms of IL-1 (IL-1α and IL-1β); IL-1 is necessary for the successful initiation of some forms of immune response [33]. IL-1 activates neutrophils and macrophages, which increases the production and release of reactive oxygen species and nitric oxide, and has been implicated in localized tissue damage [34]. Therefore, inhibition of these cytokines may contribute to a reduction in neutrophil infiltration, as well as reductions in bone and cementum destruction [7,8]. We found significant decreases in gingival TNF-α and IL-1β levels in rats treated with all test substances.

Oxidative stress associated with the pathogenesis of periodontitis has been revealed, including nitric oxide activities [6], and many antioxidants have been shown to have favorable effects on periodontitis and related alveolar bone loss [35,36,37]. MDA is an index of lipid peroxidation [38], and has been shown to increase in periodontal disease [39]. NO is produced by oxidation of l-arginine via a family of isoenzymes, and is an important mediator of a variety of physiological and pathophysiological processes [40,41]. In particular, iNOS is a distinct isoform of NOS, and can be induced in a variety of cells by pro-inflammatory agents such as endotoxins, IL-1β and TNF-α. Enhanced formation of NO following the induction of iNOS has been implicated in the pathogenesis of shock and inflammation [40,41]. Activation of iNOS and related increases of NO production occurs during periodontal disease, which leads to damage to surrounding tissues, especially the alveolar bones [6,39]. In our previous studies [7,8], Polycan and calcium gluconate treatment remarkably reduced the increase of iNOS activity and MDA levels, concomitantly inhibited the pro-inflammatory cytokines IL-β and TNF-α. Furthermore, calcium gluconate showed a decrease in H_2_O_2_-treated cells via ROS scavenging effect. Thus, mixture Polycan and calcium gluconate, Polycal was expected to act antioxidant and anti-inflammatory effect in experimental rodent model of periodontitis. In current study, we found that the increases in MDA levels and iNOS activity were smaller following treatment with all test substances. According to our results, Polycal has significant potential as an ingredient of functional foods for the treatment of periodontitis and related alveolar bone loss. Thus, Polycal can be applied as a potential functional foods or drug.

As with previous EPD studies [42,43], periodontitis leads to marked infiltration of inflammatory cells, as well as edematous changes in the gingival tissues between the upper incisors. In addition, we found evidence of absorption of the alveolar bones due to osteoclast cell activity via histopathological observations. Increases in histological scores are associated with infiltration of inflammatory cells and alveolar bone damage [4,23,44]. The infiltration of inflammatory cells, decreases in collagen-occupied regions related to edematous changes, decreases in bone volume, increases in the number of osteoclast cells and increases in the ratio OS/BS that were detected via our histomorphometrical analyses are consistent with previous reports [7,8]. These histopathological changes related to periodontitis and alveolar bone loss were reduced markedly with oral administration of IND, as well as by topical application of Polycal at all three concentrations, in which the response was concentration response trend.

## 4. Materials and Methods

### 4.1. Animals and Husbandry

Male SD (Crl:CD1) rats (6-week old upon receipt; OrientBio, Seungnam, Korea), were used following acclimatization for 10 days. Animals were allocated in polycarbonate cage at 20–25 °C, 50%–55% humidity, and 12-h light–dark cycle with free access to standard rodent chow (Samyang, Korea) and water. All laboratory animals were treated according to the national regulations for the usage and welfare of laboratory animals, and the experiments were approved by the Institutional Animal Care and Use Committee in Daegu Haany University (Gyeongsan, Gyeongbuk, Korea) (Approval No. DHU2015-035) prior to the beginning of the study. Six groups (total 40 EPD and 8 Intact rats; 8 rats in each group) were used in this experiment.

### 4.2. Preparations and Administration of Test Materials

Polycal was supplied as a 2:98 (g/g) mixture of Polycan and calcium gluconate by Aribio (Seoul, Korea). Indomethacin (IND) was purchased from Sigma-Aldrich (St. Louise, MO, USA). Solutions of distilled water containing 50, 25, and 12.5 mg/mL of Polycal were prepared, and applied topically and directly onto the ligated gingival regions (200 μL/rat) once a day for 10 continuous days from 24 h after placement of the ligations. Test materials were spread around the ligated incisor teeth using syringe. IND was dissolved in distilled water and administrated orally once a day for 10 days at 5 mL/kg (5 mg/kg) as a reference. In both Intact and experimental periodontal disease (EPD) controls, the same volume of distilled water was applied in place of the test substances.

### 4.3. Induction of EPD

EPD was induced using sterilized nylon 3-0 thread ligatures placed around the cervix of the upper left incisor. The rats were anesthetized via inhalation of 2%–3% isoflurane (Hana Pharm. Co., Hwasung, Korea) in a mixture of 70% N_2_O and 28.5% O_2_. The ligatures were knotted on the buccal side of the teeth, resulting in subgingival positioning palatinally in the supragingival position buccally. In Intact control rats, the cervix of the upper left incisor was identified, but ligatures were not placed.

### 4.4. Measurement of Bodyweight and Alveolar Bone Loss

Changes in bodyweight were measured daily from 1 day prior to ligature placement throughout the experimental period using an automatic electronic balance (Precisa Instrument, Dietikon, Switzerland). The animals were euthanized at 10 days after administration, and the maxillary bone containing the ligature placement site was excised. The anterior side of gingival tissue of ligated incisor teeth used for histological analysis, and the posterior side for biochemical analysis on the basis of the coronal section of ligated incisor teeth. The horizontal alveolar bone loss and the distance between the cusp tip and the alveolar bone was measured using a modification of the method reported by Crawford *et al.* [45], as described by Samejima *et al.* [3]. Measurements were made along the axis of the root of the upper left incisor; data are expressed as mm/rat.

### 4.5. Microbiological Analysis

The buccal gingival tissues surrounding the upper left incisor were removed, and placed in 0.3 mL of brain heart infusion (BHI) broth (Becton, Dickinson and Company, Cockeysville, MD, USA). The collected fragment was then homogenized immediately, and plated in dilutions of 1:100 and 1:1000 into blood agar (BHI agar supplemented with 5% defibrinated sheep blood and 10 μg/mL of henin/menadione; Dickinson and Company), and incubated at 37 °C for 48 h under 5% CO_2_ aerobic conditions. After 48 h of incubation, colony forming units (CFUs) were counted; data are expressed as ×10^5^ CFU/g of tissue.

### 4.6. Measurement of Myeloperoxidase Activity

The buccal gingival tissues surrounding the left incisor were removed and stored at −70 °C. To solubilize the myeloperoxidase (MPO), the material was suspended in 0.5% hexadecyltrimethyl-ammonium bromide (Gibco, Carlsbad, CA, USA) in a 50-mM potassium phosphate buffer at pH 6.0. Following homogenization in an ice bath for 15 s, samples underwent two freeze–thaw cycles. Additional buffer was added to the test tube to provide 400 μL of buffer per 15 mg of tissue for 12 min. Following centrifugation at 1000*× g* for 12 min, 0.1 mL of the supernatant was added to 2 mL of phosphate buffer, containing 0.167 mg/mL of o-dianisidine dihydrochloride (Sigma-Aldrich), distilled water and 0.0005% hydrogen peroxide, which provided a final volume of 2.1 mL per tube. The optical absorbance was measured at 460 nm using ultraviolet–visible (UV-Vis) spectrometry (OPTIZEN POP, Mecasys, Daejeon, Korea). One unit (U) of activity was defined from the degradation of 1 μM of peroxide/min at 25 °C. Data are expressed as MPO U/mg tissue.

### 4.7. Detection of IL-1β and TNF-α in Rat maxillary Gingival Tissue

Micro titer plates were coated overnight at 4 °C with antibodies against rat TNF-α or IL-1β. After blocking the plates, the homogenized tissue samples and a standard were added at various dilutions, and incubated at 4 °C for 24 h. The plates were washed three times with buffer, and then biotinylated sheep polyclonal anti-rat TNF-α or anti-rat IL-1β (Abcam, Cambridge, UK), was added to the wells. Following further incubation at room temperature for 1 h, the plates were washed and avidin-HRP (diluted 1:5000; Abcam, Cambridge, UK) was added. After 15 min, o-phenylenediamine (Sigma-Aldrich) was added, and the plates were incubated in the dark at 37 °C for 20 min. The enzyme reaction was stopped using H_2_SO_4_, and the absorbance was measured at 490 nm. Data are expressed as pg/mL.

### 4.8. MDA Measurement

Tissue samples were homogenized in a *p*H-7.4 buffer composed of 50-mM Tris-HCl, 0.1-mM EGTA and 1-mM phenylmethylsulfonyl fluoride. Aliquots of the buccal gingival tissue homogenate were added to a reaction mixture containing 8.1% sodium dodecyl sulfate (Sigma-Aldrich), 20% acetic acid, 0.8% thiobarbituric acid (Sigma-Aldrich) and distilled water. Samples were then heated to 95 °C for 1 h, and centrifuged at 3000*× g* for 10 min. The absorbance of the supernatant was measured at 650 nm using a UV-Vis spectrophotometer. Data are expressed as μM/mg of tissue.

### 4.9. iNOS Activity Measurement

Homogenates were incubated in the presence of l-[3*H*]-arginine (10 mM), NADPH (1 mM), calmodulin (30 nM), tetrahydrobiopterin (5 mM) and calcium (2 mM) at 22 °C for 30 min. Reactions were terminated by dilution with ice-cold HEPES buffer containing EGTA (2 mM) and EDTA (2 mM). Experiments were carried out in the absence of NADPH to determine the extent of l-[3*H*]-citrulline formation. Experiments in the presence of NADH (but not calcium), as well as in the presence of EGTA (5 mM), were carried out to determine the calcium-independent NOS activity. The reaction mixtures were placed in Dowex 50W (Na/form) columns, and the eluted l-[3*H*]-citrulline activity was measured using a liquid scintillation counter (Wallac, Annapoli, MD, USA). All reagents used in the iNOS activity measurements were obtained from Sigma-Aldrich. Data are expressed as fM/mg/min.

### 4.10. Histopathology

The region of the maxilla surrounding the ligature placement was fixed in 10% neutral buffered formalin. Following fixation, they were decalcified using decalcifying solution (24.4% formic acid and 0.5-M sodium hydroxide) for 5 days, during which the decalcifying solution was exchanged daily. They were then longitudinally trimmed embedded in paraffin, sectioned and stained using Hematoxylin and Eosin (H & E) according to established methods [7,8]. The histological profiles of the gingival tissues and alveolar bones were observed and compared to the Intact control. The region between the left and right incisors was analyzed using optical microscopy, and ranked using a score of 0 to 3, considering the influx of inflammatory cells, as well the integrity of the alveolar bone and cementum (Table 3). In addition, the number of infiltrated inflammatory cells per unit area (mm^2^) of gingival tissues were measured, as well as the fraction of collagen-occupied regions of the gingival areas between the first and second molars, using histomorphometrical methods with longitudinally trimmed samples using the computer-assisted image analysis software package *i*Solution FL ver 9.1 (IMT *i*-solution Inc., Vancouver, QC, Canada). We also characterized the alveolar bone volume (%/mm^2^ of alveolar bone areas), osteoclast cell number (n/mm^2^ of alveolar bone surface), and the proportion of the surface that was occupied by osteoclasts (%/mm^2^ of the alveolar bone surface) on the alveolar bone regions using *i*Solution. The histological data was analyzed one measurement per a rat, and eight rats in each group. The histopathologist was blinded to the group distribution during this analysis.

### 4.11. Statistical Analysis

All data are expressed as the mean ± standard deviation (SD) of eight rats. The variance homogeneity was examined using the Levene test. If the Levene test indicated no significant deviation from the variance homogeneity, the obtained data were analyzed using one way analysis of variance followed by least-significant differences multi-comparison tests to determine which pairs of group comparisons were significantly different. Where significant deviations from variance homogeneity were observed, the data were analyzed using Kruskal–Wallis H-test followed by the Mann–Whitney U-test to determine the specific pairs that were significant in the group comparison. Statistical analyses were implemented using SPSS for Windows (Release 14.0K, IBM SPSS Inc., Armonk, NY, USA).

## 5. Conclusions

The results of this study suggest that topical application of Polycal has significant inhibitory effects on periodontitis and related alveolar bone loss induced by EPD through the antibacterial, anti-inflammatory and anti-oxidative activities. Polycal (25 mg/mL) exhibited similar favorable inhibitory effects to IND (5 mg/kg) in terms of anti-inflammatory and antioxidant responses; however, treatment with IND did not influence gingival bacterial proliferation, and resulted in a significant decrease in bodyweight compared with the EPD control. Our previous study demonstrated that the oral administration of Polycal showed the significantly anti-periodontitis. Therefore, Polycal has significant potential as an ingredient of functional foods for the treatment of periodontitis and related alveolar bone loss.

## Figures and Tables

**Table 1:** Body weight gains after 10 days of continuous treatment in Intact or EPD Rats.

	Items	Body Weights (g)				Body Weight Gains [B − A]
Groups		Before Ligation	At Ligation	At Initiation of Test Article Treatment [A]	At Last Administration of Test Article [B]	
Controls						
Intact		250.13 ± 11.99	221.00 ± 14.20	248.50 ± 13.10	279.63 ± 11.83	31.13 ± 4.16
EPD		250.63 ± 11.76	222.75 ± 12.94	241.13 ± 10.83	247.00 ± 10.81 ^a^	5.88 ± 4.70 ^a^
Reference						
IND		249.75 ± 14.03	221.25 ± 14.91	242.00 ± 16.37	225.63 ± 14.99 ^a,c^	16.38 ± 5.10 ^a,c^
Polycal concentrations						
H: 50 mg/mL		250.38 ± 17.52	220.88 ± 17.72	242.00 ± 15.69	265.75 ± 16.82 ^b,c^	23.75 ± 4.80 ^a,c^
M: 25 mg/mL		250.38 ± 14.38	222.63 ± 16.48	241.75 ± 14.10	262.00 ± 8.78 ^a,d^	20.25 ± 6.30 ^a,c^
L: 12.5 mg/mL		250.25 ± 14.92	222.13 ± 16.03	242.88 ± 13.55	259.50 ± 9.55 ^a^	16.63 ± 6.57 ^a,c^

Values are expressed mean ± S.D. of eight rats, g. EPD = Experimental periodontal diseases induced by ligature placement around the cervix of upper left incisor teeth; Polycal = Polycan and calcium gluconate 2:98 (g/g) mixture, which was topically applied to ligated gingival regions, once a day for 10 days (200 μL/rats); IND = Indomethacin, which was orally administered at 5 mg/kg dose level in a volume of 5 mL/kg, once a day for 10 days. ^a^ *p <* 0.01 and ^b^ *p <* 0.05 as compared with Intact control; ^c^ *p <* 0.01 and ^d^ *p <* 0.05 as compared with EPD control.

**Table 2:** Histomorphometrical analysis of maxillary regions around ligation placement—gingival and alveolar bone areas in Intact or EPD Rats.

	Items	Gingival Areas		Alveolar Bone Areas		
Groups		Inflammatory cells (Cells/mm^2^)	Collagen Percentages (%/mm^2^)	Alveolar Bone volume (%)	Osteoclast Cell (cells/mm)	OS/BS (%)
Controls						
Intact		9.38 ± 3.74	68.06 ± 11.52	65.68 ± 10.19	5.88 ± 1.73	1.74 ± 0.91
EPD		1110.00 ± 265.89 ^a^	21.66 ± 10.06 ^a^	33.17 ± 10.29 ^a^	38.38 ± 10.39 ^a^	35.21 ±10.36 ^a^
Reference						
IND		226.38 ± 73.92 ^a,b^	49.65 ± 11.52 ^a,b^	49.70 ± 10.85 ^a,b^	25.38 ± 5.01 ^a,b^	20.05 ± 4.18 ^a,b^
Polycal concentrations						
H: 50 mg/mL		137.00 ± 45.42 ^a,b^	61.28 ± 12.05 ^b^	56.66 ± 11.61 ^b^	15.38 ± 5.18 ^a,b^	11.69 ± 3.65 ^a,b^
M: 25 mg/mL		234.25 ± 76.65 ^a,b^	50.13 ± 12.77 ^a,b^	50.01 ± 11.16 ^a,b^	23.88 ± 4.97 ^a,b^	18.95 ± 4.89 ^a,b^
L: 12.5 mg/mL		488.75 ± 109.65 ^a,b^	42.33 ± 15.22 ^a,b^	47.44 ± 7.59 ^a,b^	26.88 ± 5.99 ^a,b^	22.66 ± 4.59 ^a,b^

Values are expressed mean ± S.D. of eight rats, g. EPD = Experimental periodontal diseases induced by ligature placement around the cervix of upper left incisor teeth; Polycal = Polycan and calcium gluconate 2:98 (g/g) mixture, which was topically applied to ligated gingival regions, once a day for 10 days (200 μL/rats); IND = Indomethacin, which was orally administered at 5 mg/kg dose level, once a day for 10 days; OS/BS = the percentages of osteoclast cells occupied regions on the alveolar bone surface. ^a^ *p <* 0.01 as compared with Intact control; ^b^ *p <* 0.01 as compared with EPD control.

**Table 3:** The Histological Scores of EPD Used in This Study

Scores	Remarks
0	Absence or only a discrete cellular infiltration (inflammatory cell infiltration is sparse and restricted to the region of the marginal gingival), preserved alveolar process and cementum
1	Moderate cellular infiltration (inflammatory cellular infiltration present all over the insert gingival), some but minor alveolar process resorption and Intact cementum
2	Accentuated cellular infiltration (inflammatory cellular infiltration present in both gingival and periodontal ligament), accentuated degradation of the alveolar process and partial destruction of cementum
3	Accentuated cellular infiltrate, complete resorption of the alveolar process and severe destruction of cementum
Max = 3	

EPD = Experimental periodontal diseases induced by ligature placement around the cervix of upper left incisor teeth. Modified from Menezes *et al.* [5].

## Abbreviations

BHI	Brain heart infusion broth
EPD	Experimental periodontitis
GLP	Good Laboratory Practice
HE	Hematoxylin-eosin
HTAB	Hexadecyltrimethyl-ammonium bromide
IL	Interleukin
IND	Indomethacin, reference
iNOS	Inducible nitric oxide synthase
MDA	Malondialdehyde
MPO	Myeloperoxidase
NSAIDs	Non-steroid anti-inflammatory drugs
OS/BS	Percentages of osteoclast cells occupied regions on the alveolar bone surface
PMNs	Polymorphneutrophils
Polycal	Polycan and calcium gluconate 2:98 (g/g) mixture, test materials
SD	Standard deviation
SPF	Specific pathogen free
TNF	Tumor necrosis factor
VAF	Virus antibody free

## References

[1] Chambrone L.A., Chambrone L. (2006). Tooth loss in well-maintained patients with chronic periodontitis during long-term supportive therapy in Brazil. J. Clin. Periodontol.

[2] Sallay K., Sanavi F., Ring I., Pham P., Behling U.H., Nowotny A. (1982). Alveolar bone destruction in the immunosuppressed rat. J. Periodontal Res..

[3] Samejima Y., Ebisu S., Okada H. (1990). Effect of infection with *Eikenella corrodens* on the progression of ligature-induced periodontitis in rats. J. Periodontal Res..

[4] Botelho M.A., Rao V.S., Carvalho C.B., Bezerra-Filho J.G., Fonseca S.G., Vale M.L., Montenegro D., Cunha F., Ribeiro R.A., Brito G.A. (2007). *Lippia sidoides* and *Myracrodruon urundeuva* gel prevents alveolar bone resorption in experimental periodontitis in rats. J. Ethnopharmacol..

[5] Menezes A.M., Rocha F.A., Chaves H.V., Carvalho C.B., Ribeiro R.A., Brito G.A. (2005). Effect of sodium alendronate on alveolar bone resorption in experimental periodontitis in rats. J. Periodontol.

[6] Lohinai Z., Benedek P., Fehér E., Györfi A., Rosivall L., Fazekas A., Salzman A.L., Szabó C. (1998). Protective effects of mercaptoethylguanidine, a selective inhibitor of inducible nitric oxide synthase, in ligature-induced periodontitis in the rat. Br. J. Pharm..

[7] Ku S.K., Cho H.R., Sung Y.S., Kang S.J., Lee Y.J. (2011). Effects of calcium gluconate on experimental periodontitis and alveolar bone loss in rats. Basic Clin. Pharmacol. Toxicol.

[8] Kim Y.S., Kang S.J., Kim J.W., Cho H.R., Moon S.B., Kim K.Y., Lee H.S., Han C.H., Ku S.K., Lee Y.J. (2012). Effects of Polycan, α β-glucan, on experimental periodontitis and alveolar bone loss in Sprague-Dawley rats. J. Periodontal Res..

[9] Seo H.P., Kim J.M., Shin H.D., Kim T.K., Chang H.J., Park B.R., Lee J.W. (2002). Production of-1, 3/1, 6-glucan by *Aureobasidium pullulans* SM-2001. Korean J. Bitechnol. Bioeng..

[10] Song H.B., Park D.C., Gyung M.D., Hwang S.L., Lee W.K., Kang H.S., Park B.R., Jang H.J., Son C.W., Park E.K. (2006). Effect of exopolymers of *Aureobasidium pullulans* on improving osteoporosis induced in ovariectomized mice. J. Microbiol. Biotechnol..

[11] Shin H.D., Yang K.J., Park B.R., Son C.W., Jang H.J., Ku S.K. (2007). Antiosteoporotic effect of Polycan, b-glucan from *Aureobasidium*, in ovariectomized osteoporotic mice. Nutrition.

[12] Kim H.D., Cho H.R., Moon S.B., Shin H.D., Yang K.J., Park B.R., Jang H.J., Kim L.S., Lee H.S., Ku S.K. (2007). Effects of β-glucan from *Aureobasidium pullulans* on acute inflammation in mice. Arch. Pharm. Res..

[13] Kim H.D., Cho H.R., Moon S.B., Shin H.D., Yang K.J., Park B.R., Jang H.-J., Kim L.S., Lee H.S., Ku S.K. (2006). Effect of exopolymers from *Aureobasidium pullulans* on formalin-induced chronic paw inflammation in mice. J. Microbiol. Biotechnol..

[14] Ku S.K., Lee Y.J., Lee S.D., Cho H.R., Moon S.B., Kim K.Y., Kwon Y.S., Kim J.W. (2012). Nephroprotective effect of POLYCAN on acute renal failure induced by cisplatin in rats. ISRN. Vet. Sci..

[15] Hendry J.A., Jeansonne B.G., Dummett C.O., Burrell W. (1982). Comparison of calcium hydroxide and zinc oxide and eugenol pulpectomies in primary teeth of dogs. Oral. Surg. Oral. Med. Oral. Pathol..

[16] Piller N.B. (1990). Assessment of the anti-inflammatory action of calcium dobesilate. Effect on macrophages attaching to subcutaneously implanted coverslips in guinea pigs. Arzneimittelforschung.

[17] Smith M.M., Ghosh P., Numata Y., Bansal M.K. (1994). The effects of orally administered calcium pentosan polysulfate on inflammation and cartilage degradation produced in rabbit joints by intraarticular injection of a hyaluronate-polylysine complex. Arthritis Rheum..

[18] Karnad A., Patil P., Majagi S. (2006). Calcium enhances antiinflammatory activity of aspirin in albino rats. Indian J. Pharmacol..

[19] Sohn K.C., Kang S.J., Kim J.W., Kim K.Y., Ku S.K., Lee Y.J. (2013). Effects of Calcium Gluconate, a Water Soluble Calcium Salt on the Collagen-Induced DBA/1J Mice Rheumatoid Arthritis. Biomol. Ther..

[20] Choi J.S., Kim J.W., Kim K.Y., Cho H.R., Choi I.S., Ku S.K. (2014). Antiosteoporotic effects of Polycan in combination with calcium lactate-gluconate in ovariectomized rats. Exp. Ther. Med..

[21] Kim J.W., Choi J.S., Ha Y.M., Choi I.S., Kim K.Y., Cho H.R., Rha C.H., Ku S.K. (2013). Single oral dose toxicity test of polycalcium, a mixed composition of polycan and calcium lactate-gluconate 1:9 (G/G) in SD rat. Pak. J. Pharm. Sci..

[22] Lee W.H., Kim K.H., Kang S.J., Lee Y.J., Ku S.K. (2014). Synergic Effects of Mixed Formula Consisted of Polycan and Calcium-gluconate on the Experimental Periodontitis and Alveolar Bone Loss in Rats. J. Soc. Prev. Korean Med..

[23] Azoubel M.C., Menezes A.M., Bezerra D., Oriá R.B., Ribeiro R.A., Brito G.A. (2007). Comparison of etoricoxib and indomethacin for the treatment of experimental periodontitis in rats. Braz. J. Med. Biol. Res..

[24] Ximenez-Fyvie L.A., Almaguer-Flores A., Jacobo-Soto V., Lara-Cordoba M., Moreno-Borjas J.Y., Alcantara-Maruri E. (2006). Subgingival microbiota of periodontally untreated Mexican subjects with generalized aggressive periodontitis. J. Clin. Periodontol.

[25] Hetland G., Sandven P. (2002). β-1,3-Glucan reduces growth of *Mycobacterium tuberculosis* in macrophage cultures. FEMS Immunol. Med. Microbiol..

[26] Dobšíková R., Blahová J., Mikuliková I., Modrá H., Prášková E., Svobodová Z., Skorič M., Jarkovský J., Siwicki A.K. (2013). The effect of oyster mushroom b-1.3/1.6-D-glucan and oxytetracycline antibiotic on biometrical, haematological, biochemical, and immunological indices, and histopathological changes in common carp (*Cyprinus carpio* L.). Fish. Shellfish. Immunol..

[27] Sen I.K., Mandal A.K., Chakraborti S., Dey B., Chakraborty R., Islam S.S. (2013). Green synthesis of silver nanoparticles using glucan from mushroom and study of antibacterial activity. Int. J. Biol. Macromol..

[28] Liu H., Pope R.M. (2004). Phagocytes: mechanisms of inflammation and tissue destruction. Rheum. Dis. Clin. N. Am..

[29] Zimmerman B.J., Grisham M.B., Granger D.N. (1990). Role of oxidants in ischemia/reperfusion-induced granulocyte infiltration. Am. J. Physiol..

[30] Işeri S.O., Sener G., Yüksel M., Contuk G., Cetinel S., Gedik N., Yegen B.C. (2005). Ghrelin against alendronate-induced gastric damage in rats. J. Endocrinol..

[31] Samira S., Ferrand C., Peled A., Nagler A., Tovbin Y., Ben-Hur H., Taylor N., Globerson A., Lapidot T. (2004). Tumor necrosis factor promotes human T-cell development in nonobese diabetic/severe combined immunodeficient mice. Stem. Cells.

[32] Isaacs A., Tizard I.R. (1995). Lymphokines and cytokines. Immunology, an Introduction.

[33] Unanue E.R., Tizard I.R. (1995). The Mononuclear-Phagocytic sytem. Immunology, an Introduction.

[34] Assuma R., Oates T., Cochran D., Amar S., Graves D.T. (1998). IL-1 and TNF antagonists inhibit the inflammatory response and bone loss in experimental periodontitis. J. Immunol..

[35] Di Paola R., Mazzon E., Zito D., Maiere D., Britti D., Genovese T., Cuzzocrea S. (2005). Effects of Tempol, a membrane-permeable radical scavenger, in a rodent model periodontitis. J. Clin. Periodontol.

[36] Cai X., Li C., Du G., Cao Z. (2008). Protective effects of baicalin on ligature-induced periodontitis in rats. J. Periodontal Res..

[37] Toker H., Ozdemir H., Eren K., Ozer H., Sahin G. (2009). *N*-Acetylcysteine, a thiol antioxidant, decreases alveolar bone loss in experimental periodontitis in rats. J. Periodontol..

[38] Cuzzocrea S., Zingarelli B., Hake P., Salzman A.L., Szabó C. (1998). Antiinflammatory effects of mercaptoethylguanidine, a combined inhibitor of nitric oxide synthase and peroxynitrite scavenger, in carrageenan-induced models of inflammation. Free Radic. Biol. Med..

[39] Di Paola R., Marzocco S., Mazzon E., Dattola F., Rotondo F., Britti D., De Majo M., Genovese T., Cuzzocrea S. (2004). Effect of aminoguanidine in ligature-induced periodontitis in rats. J. Dent. Res..

[40] Szabó C. (1995). Alterations in nitric oxide production in various forms of circulatory shock. New Horiz..

[41] Southan G.J., Szabó C. (1996). Selective pharmacological inhibition of distinct nitric oxide synthase isoforms. Biochem. Pharmacol..

[42] Bezerra J.P., da Silva L.R., de Alvarenga Lemos V.A., Duarte P.M., Bastos M.F. (2008). Administration of high doses of caffeine increases alveolar bone loss in ligature-induced periodontitis in rats. J. Periodontol..

[43] Holanda Pinto S.A., Pinto L.M., Cunha G.M., Chaves M.H., Santos F.A., Rao V.S. (2008). Anti-inflammatory effect of a, b-Amyrin, a pentacyclic triterpene from *Protium heptaphyllum* in rat model of acute periodontitis. Inflammopharmacology.

[44] Leitão R.F., Ribeiro R.A., Chaves H.V., Rocha F.A., Lima V., Brito G.A. (2005). Nitric oxide synthase inhibition prevents alveolar bone resorption in experimental periodontitis in rats. J. Periodontol..

[45] Crawford J.M., Taubman M.A., Smith D.J. (1978). The natural history of periodontal bone loss in germfree and gnotobiotic rats infected with periodontopathic microorganisms. J. Periodontal Res..

